# Efficacy of osimertinib and the role of sequential liquid biopsy in patients diagnosed with NSCLC harboring EGFR and BRAF mutations at baseline: insights from two case reports

**DOI:** 10.3389/fonc.2024.1363069

**Published:** 2024-03-11

**Authors:** Loc Carlo Bao, Alessia Padovan, Andrea Boscolo Bragadin, Lorenzo Calvetti, Valentina Guarneri, Laura Bonanno, Stefano Indraccolo

**Affiliations:** ^1^ Department of Surgery, Oncology and Gastroenterology, University of Padua, Padua, Italy; ^2^ Basic and Translational Oncology, Veneto Institute of Oncology IOV – IRCCS, Padua, Italy; ^3^ Department of Oncology, Azienda ULSS 8 Berica, San Bortolo General Hospital, Vicenza, Italy; ^4^ Medical Oncology 2, Veneto Institute of Oncology IOV – IRCCS, Padua, Italy

**Keywords:** liquid biopsy, EGFR, NSCLC, BRAF, co-mutation, NGS, osimertinib

## Abstract

Epidermal Growth Factor Receptor (EGFR) and B-Raf (BRAF) mutations are two of the most important drivers identified in non-small-cell lung cancer (NSCLC). This report highlights two cases of patients diagnosed with metastatic NSCLC bearing concurrent EGFR and BRAF mutations at baseline and treated with osimertinib as first-line treatment. Molecular profiling was conducted in the tissue and plasma at the time of initial diagnosis, and subsequent repeated liquid biopsy examinations were planned after 10 days, 28 days, and at the time of radiological progression in the frame of the prospective translational study REM. These cases suggest that osimertinib may maintain its therapeutic effectiveness even in patients presenting with a baseline BRAF co-mutation. Notably, radiological responses align with liquid biopsy observations: in both instances, follow-up liquid biopsies indicate the clearance of EGFR-mutated circulating tumor DNA (ctDNA).

## Introduction

In the last two decades, the introduction of targeted therapies for oncogene-addicted diseases changed the natural history of non-small-cell lung cancer (NSCLC) (1–4). Oncogene addiction is a term used to describe the reliance of certain neoplasms on a single activated oncogenic protein or pathway in order to maintain malignant properties and serves as a rationale for target therapy (5).

Currently, several targeted therapies are approved for patients with oncogene-addicted tumors. EGFR and BRAF are two of the most important driver mutations, accounting for an overall prevalence of 32% and 1%–3% of NSCLC cases, respectively (6, 7).

Following the results of the FLAURA trial, osimertinib, a third generation TKI, became the standard first-line treatment for EGFR mutated metastatic NSCLC (mNSCLC) (1).

Nevertheless, the magnitude of clinical benefit is heterogeneous, and new-combination therapeutic strategies recently demonstrated to be associated with improved progression-free survival (8, 9). While reliable predictive markers are not yet available for clinical practice, the presence of co-mutations, such as p53 and PIK3CA, was found to be associated with limited clinical benefit, albeit little is known about the role of BRAF co-mutations in this particular setting (10). Interestingly, BRAF mutations have been described as a possible mechanism of acquired resistance to EGFR-TKIs (11).

Additionally, even though oncogene driver mutations are generally mutually exclusive (12), the presence of two actionable driver mutations represents a significant clinical challenge in terms of selection of treatment and management.

Here, we present two cases of patients diagnosed with mNSCLC harboring both EGFR and BRAF mutations at baseline. The patients were treated with first-line osimertinib, and tumor molecular profiling was performed in the tissue and plasma at diagnosis and monitored through repeated liquid biopsies at different time points according to the schedule of the REM clinical study. REM is an ongoing multicentric, prospective, observational clinical study that enrolls EGFR-mutated NSCLC patients receiving first-line osimertinib aiming to identify concomitant genetic alterations in plasma at baseline and at progression and to monitor EGFR mutation in plasma to correlate it with the radiological response and the outcome. In this setting, plasma samples were collected before the initiation of treatment and then after 10 days and 28 days of treatment. Three different tests were used to analyze patients’ cfDNA: a real-time PCR technique (Cobas EGFR mutation test V2, Roche) and two next-generation sequencing (NGS) tests that included the main EGFR and BRAF mutations (Avenio ctDNA Expanded kit, Roche, and PSS Solid Cancer IVD kit, Sysmex). The study design and informed consent were submitted and approved by the local Ethics Committee.

## Case description

### Case 1

The first case that we report here is one of an 81-year-old female Caucasian never-smoker patient.

In February 2023, she experienced dyspnea and underwent a CT scan that demonstrated the presence of a left inferior lobe lesion, multiple pleural homolateral nodules, pleural effusion, and a single cerebral lesion, a benign meningioma, that had already been reported in her previous clinical history. Histological diagnosis was obtained by CT-guided percutaneous needle biopsy and was compatible with lung adenocarcinoma. Real-time PCR (RT-PCR) testing in the tissue revealed the presence of both BRAF V600E mutation and EGFR exon 19 deletion (ex19del). No quantitative data of mutation frequency was available, making it impossible to distinguish whether these mutations are clonal or subclonal. Liquid biopsy at baseline confirmed the presence of EGFR ex19del at low levels in cfDNA, with correspondence among the results of three different methods used: Cobas EGFR mutation test V2 ISQ 9.79; Avenio ctDNA Expanded kit VAF 0.34%; Sysmex PSS Solid Cancer IVD kit VAF 0.25% (16 mutant molecules—MM). Conversely, the BRAF V600E mutation was not detected by both NGS tests in plasma.

The patient was in good clinical condition (ECOG PS 0) and had no relevant medical history, apart from controlled hypertension. Considering clinical staging, histological diagnosis, lack of smoking history, and molecular profile, the patient started on osimertinib, and close clinical and radiological monitoring was performed.

Subsequent radiological tumor assessments were performed according to clinical practice, and best radiological response was stable disease according to RECIST 1.1. The treatment was well tolerated, and the only adverse event recorded was a G1 platelet count decrease (according to CTCAE 5.0).

Liquid biopsy testing was performed initially by Cobas EGFR mutation test V2 RT-PCR at different time points, revealing clearance of the EGFR mutation coherent with the clinical response to treatment; indeed, cfDNA samples from T1 to T5 resulted in non-mutated EGFR. Consistently, analysis of serial cfDNA samples by Sysmex PSS Solid Cancer IVD kit detected minimal residual molecular disease at T1 (VAF, 0.046%; 4.22 MM) and T2 (VAF, 0.029%; 2.34 MM) with a kinetics showing gradual reduction in the EGFR mutation during treatment. Indeed, in the following time points (T3–T4–T5), NGS analysis indicated complete clearance of the mutation in plasma. Notably, the BRAF mutation found in tissue biopsy was not detected in any of the cfDNA samples analyzed by Sysmex PSS Solid Cancer IVD kit.

After 12 months, the patient is still on treatment with osimertinib, and she is maintaining a good clinical and radiological response. Further plasma monitoring and re-characterization in the tissue and plasma at the time of progression has been planned.

### Case 2

The second case is that of a 77-year-old Caucasian woman.

Following the onset of dyspnea in July 2022, she underwent a CT scan showing bilateral lung nodules and left pleural effusion for which she underwent left thoracentesis with immediate clinical benefit. A CT-scan-guided needle biopsy of one of the lung nodules performed for diagnostic purposes enabled to make the histological diagnosis of lung adenocarcinoma, with no evidence of extra-thoracic disease. Molecular profiling on the tissue revealed the presence of EGFR L858R and BRAF E501K mutations, without available variant allele fraction information. NGS testing of cfDNA at baseline confirmed the presence of both mutations. Specifically, Avenio ctDNA Expanded kit detected EGFR L858R with VAF = 0.35% and Sysmex PSS Solid Cancer IVD kit detected the same mutation with VAF = 0.15%. Cobas EGFR mutation test V2 RT-PCR analysis was in line with these results, revealing EGFR L858R mutation with ISQ = 5.99. BRAF mutation E501K was confirmed in plasma only by Avenio ctDNA Expanded kit (VAF 0.31%), since this mutation is not covered by Sysmex PSS Solid Cancer IVD kit. The similarity in VAF detected for EGFR and BRAF mutations may suggest the clonal nature of the BRAF mutation.

Considering the good performance status, clinical staging, and molecular characterization of disease, the patient started her first-line systemic treatment with osimertinib.

RT-PCR testing for EGFR was then performed on liquid biopsy at time points T1 and T2, revealing clearance of EGFR mutation right after 10 days since the start of systemic treatment. NGS monitoring by Sysmex PSS Solid Cancer IVD kit was performed only at T2 due to insufficient amount of cfDNA available at T1 and confirmed the clearance of EGFR mutation. BRAF mutation monitoring was not possible because the E501K mutation is not included in the Sysmex PSS Solid Cancer IVD panel.

The first radiological assessment revealed a partial response according to RECIST 1.1 with a 70% reduction in the sum of target lesions. At the time of writing, 17 months after diagnosis, the patient is still on treatment and maintains both radiological response and clinical benefit.

### Timeline

**Figure 1:**
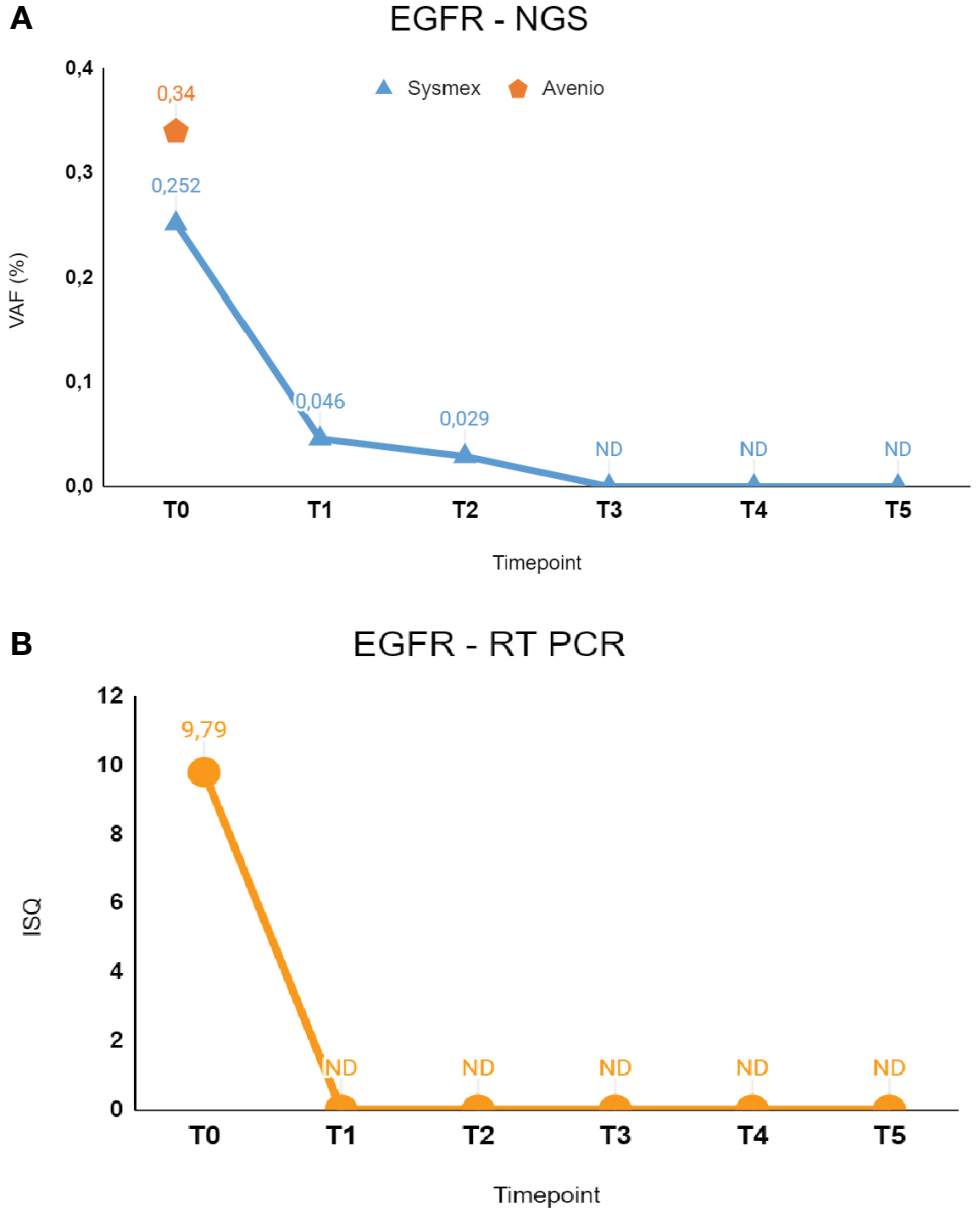
The figure shows longitudinal monitoring of EGFR ex19del mutation in the patient described as Case 1. **(A)** Results of NGS analysis: baseline T0 shows VAF values from both Avenio ctDNA Expanded kit and Sysmex PSS Solid Cancer IVD kit analysis. The data reported from T1 to T5 indicate the VAF values detected by Sysmex PSS Solid Cancer IVD kit panel after the start of treatment with osimertinib. **(B)** Monitoring of EGFR mutation by RT-PCR Cobas EGFR Mutation Test V2. Time-points were the same as in **(A)**. T0: 02/09/2023; T1: 02/17/2023; T2: 03/09/2023; T3: 04/06/2023; T4: 06/09/2023; T5: 08/24/2023. VAF, Variant Allele Fraction; ISQ, Semi-Quantitative Index.

**Figure 2:**
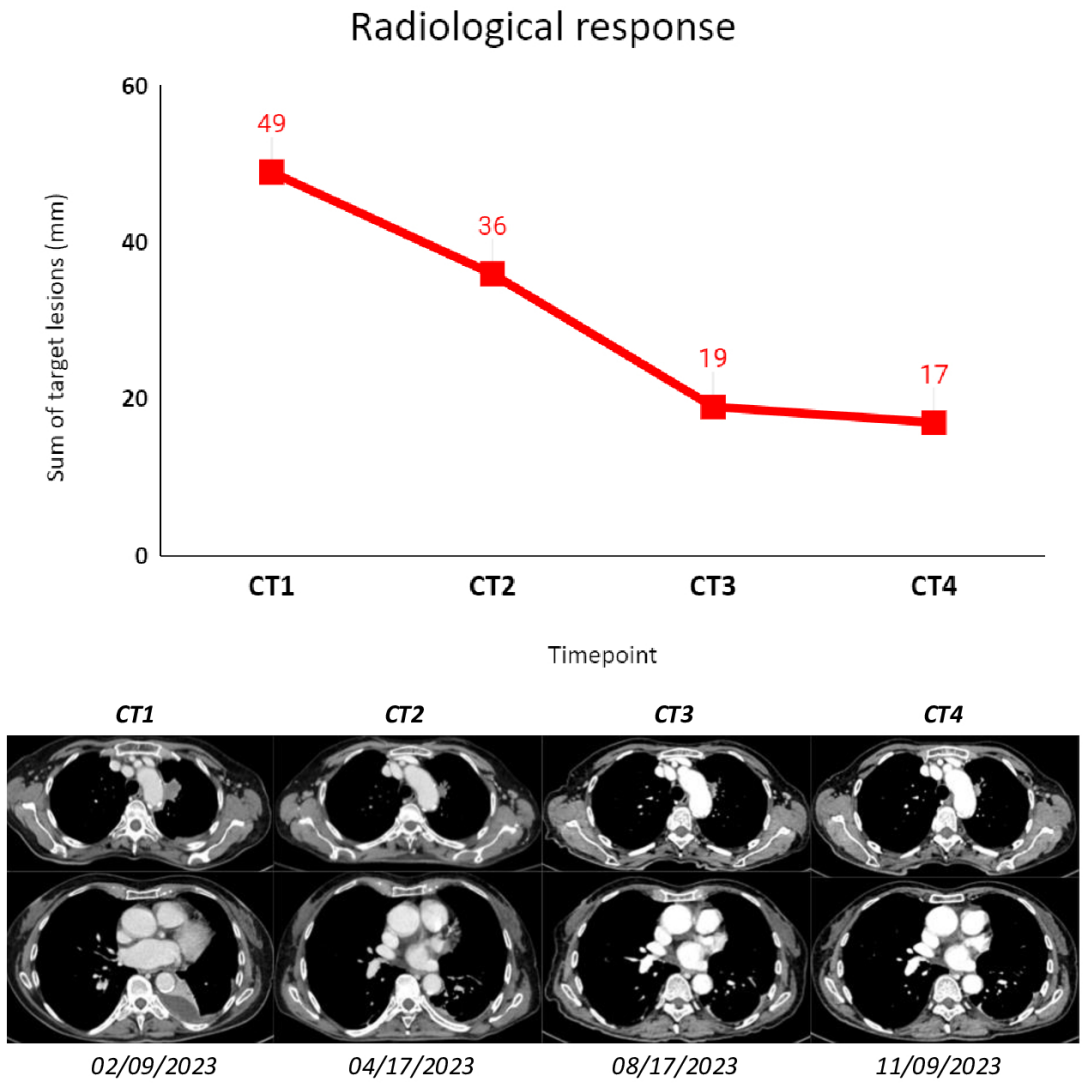
The figure shows subsequent radiological assessments (CT scans) that were performed according to clinical practice for the patient described as Case 1. The sum of target lesions was assessed following RECIST 1.1. CT1: 02/09/2023; CT2: 04/17/2023; CT3: 08/17/2023; CT4: 11/09/2023.

**Figure 3:**
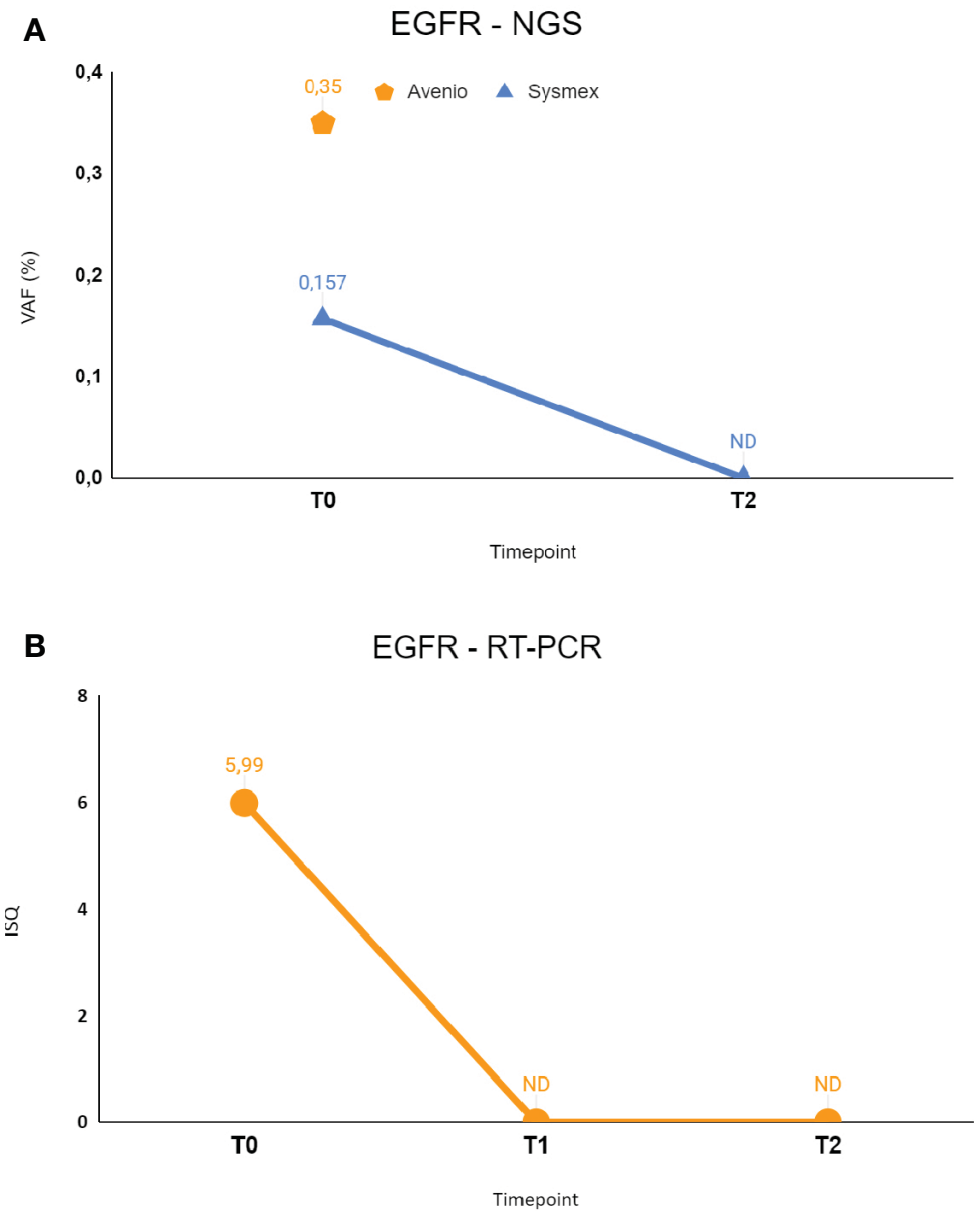
The figure shows longitudinal monitoring of EGFR ex19del mutation in the patient described as Case 2. **(A)** Results of NGS analysis: baseline T0 shows VAF values from both Avenio ctDNA Expanded kit and Sysmex PSS Solid Cancer IVD kit analysis. The data reported as T2 indicate the VAF value detected by Sysmex PSS Solid Cancer IVD kit panel after the start of treatment with osimertinib. **(B)** Monitoring of EGFR mutation by RT-PCR Cobas EGFR Mutation Test V2. T0 and T2 were the same as in **(A)**, T1 was the first sample after the start of treatment with osimertinib. T0: 09/06/2022; T1: 09/14/2022; T2: 09/26/2022. VAF, Variant Allele Fraction; ISQ, Semi-Quantitative Index.

**Figure 4:**
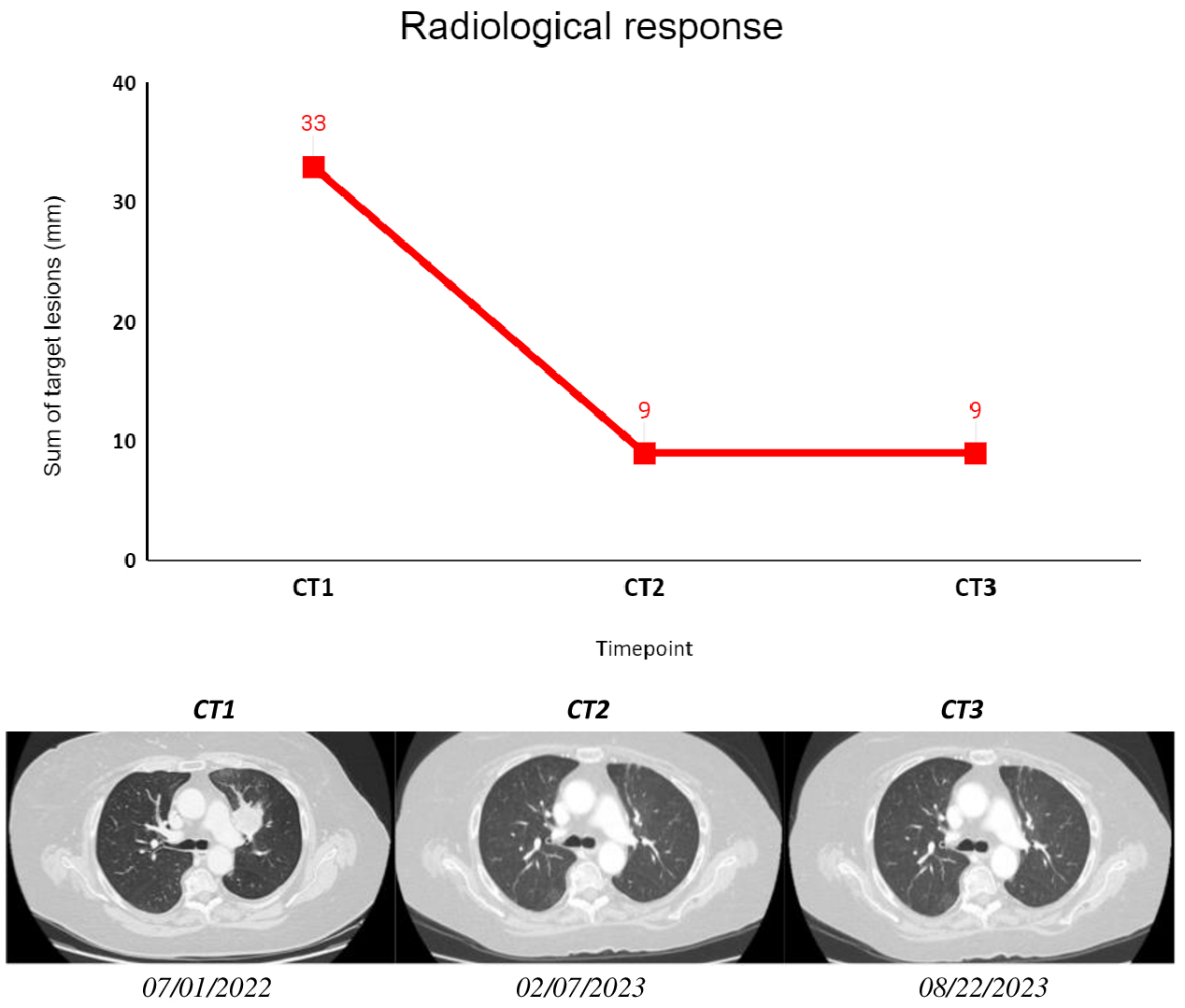
The figure shows subsequent radiological assessments (CT scans) that were performed according to clinical practice for the patient described as Case 2. The sum of target lesions was assessed following RECIST 1.1. CT1: 07/01/2022; CT2: 02/07/2023; CT3: 08/22/2023.

The results of longitudinal liquid biopsies and radiological assessment for the patient described in case 1 are reported in **Figures 1**, **2**, while those for case 2 are shown in **Figures 3**, **4**.

### Molecular diagnostic assessment

According to the REM protocol, cfDNA samples were collected before starting treatment (T0) and after 10 (T1) and 28 days (T2).

The patient described in case 1 displayed both EGFR and BRAF mutations at tissue biopsy, so she was monitored through longitudinal liquid biopsy at every medical appointment, resulting in a total of six samples (from T0 to T5) being evaluated. The first three blood samples were taken as described, while the additional three samples were collected approximately every 2 months. Regarding case 2, cfDNA samples were collected as planned before and after early time points from osimertinib administration (T0–T1–T2).

We used three methods to test patient’s cfDNA: Cobas EGFR mutation test V2 (Roche), Avenio ctDNA Expanded kit (Roche), and PSS Solid Cancer IVD kit (Sysmex).

The first method is a RT-PCR technique developed to detect EGFR mutations present in exons 18,19, 20, and 21, and it was performed for all samples of plasma collected.

The second and third methods are NGS tests, and both panels include EGFR and BRAF most common mutations as targets. Avenio ctDNA Expanded kit, a relatively large panel of 77 genes, was used only at diagnosis, while the Sysmex PSS Solid Cancer IVD kit, which includes five genes in specific hotspots, guarantees higher sensitivity, and this kind of targeted approach was ideal to monitor the minimal residual disease.

All prepared libraries were sequenced on an Illumina Nextseq 550 instrument with high output kit (300 cycles) in pair-end mode (151×2) for Avenio ctDNA Expanded kit and the mid output kit (150 cycles) in single-end mode (150 cycles) for Sysmex PSS Solid Cancer IVD kit.

## Discussion

These two cases suggest that osimertinib might maintain its efficacy even in patients with a BRAF co-mutation at baseline. Since the patients are respectively at their 12th and 17th month on treatment with persistence of clinical benefit and clearance of EGFR mutation in plasma, we suggest that patients carrying BRAF co-mutations might represent a different molecular subset of EGFR mutated patients, when compared with other co-mutations and in particular p53 and KRAS co-mutations (13, 14).

Radiological response was accompanied by liquid biopsy findings: for both cases, subsequent liquid biopsies showed a clearance in the EGFR-mutated cfDNA. Curiously, for the first patient, BRAF V600E was only found in tissue NGS analysis. This could be attributed to the fact that the patient only presented with intrathoracic disease with limited ctDNA shedding. Alternatively, considering that EGFR mutation tested positive in plasma at relatively low levels, we might speculate that the BRAF V600E was subclonal in the tumor of this patient, and therefore, it was missed by cfDNA testing because it was under the detection limit of the NGS assays used. This case clearly illustrates the potential of liquid biopsy in shedding light on tumor heterogeneity and differentiating biological significance of two driver alterations.

Notably, the second patient carried a non-V600 BRAF mutation located in the kinase domain of the protein (15). However, this mutation is currently considered inconclusive because there is conflicting and/or weak data describing the biological significance of the BRAF E501K mutation. *In vitro* studies have demonstrated that this mutation might be inactivating as measured by decreased BRAF kinase activity in a cell line with a second BRAF mutation compared to controls (16). However, another pre-clinical study found increased downstream pathway output compared to wild type (17). Nevertheless, it is still relevant to emphasize that, in this clinical case, this mutation does not seem to have a negative impact on the efficacy of osimertinib.

According to the REM protocol, RT-PCR and NGS cfDNA testing are planned also at radiological progression of disease in order to further investigate possible acquired resistance mechanism. It will be interesting to see whether BRAF mutations will be among these, since combination therapies with anti-EGFR and anti-BRAF TKIs have been described in this setting (18–20).

These two cases demonstrate that liquid biopsy can have an important role in monitoring patients during treatment, showing that molecular response is associated with clinical response and can be evaluated before radiological assessment. In addition, specifically in the context of co-mutations, further data collections are awaited to understand its potential role in unveiling tumor heterogeneity and differential role of concomitant genetic alterations.

In the end, we conclude that a molecular survey of patients’ plasma has clinical validity in this setting and can aid to follow EGFR TKI effects also in the rare event of BRAF co-mutations.

## Data availability statement

The data presented in the study are deposited in the ENA (European Nucleotide Archive) repository, accession number ERP158121.

## Ethics statement

The studies involving humans were approved by ethics committee for clinical trials, Veneto Institute of Oncology IOV – IRCCS, Padua, Italy. The studies were conducted in accordance with the local legislation and institutional requirements. The participants provided their written informed consent to participate in this study. Written informed consent was obtained from the individual(s) for the publication of any potentially identifiable images or data included in this article.

## Author contributions

LCB: Data curation, Investigation, Writing – original draft, Writing – review & editing. AP: Data curation, Investigation, Writing – original draft, Writing – review & editing. ABB: Data curation, Investigation, Writing – review & editing. LC: Writing – review & editing, Investigation. VG: Writing – review & editing, Supervision. LB: Conceptualization, Supervision, Writing – review & editing, Methodology. SI: Conceptualization, Supervision, Writing – review & editing, Methodology.

## References

[1] Ramalingam SS Vansteenkiste J Planchard D Cho BC Gray JE Ohe Y . Overall survival with osimertinib in untreated, EGFR-mutated advanced NSCLC. N Engl J Med. (2020) 382:41–50. doi: 10.1056/NEJMoa1913662 31751012

[2] Mok T Camidge DR Gadgeel SM Rosell R Dziadziuszko R Kim DW . Updated overall survival and final progression-free survival data for patients with treatment-naive advanced ALK-positive non-small-cell lung cancer in the ALEX study. Ann Oncol Off J Eur Soc Med Oncol. (2020) 31:1056–64. doi: 10.1016/j.annonc.2020.04.478 32418886

[3] Shaw AT Riely GJ Bang YJ Kim DW Camidge DR Solomon BJ . Crizotinib in ROS1-rearranged advanced non-small-cell lung cancer (NSCLC): updated results, including overall survival, from PROFILE 1001. Ann Oncol Off J Eur Soc Med Oncol. (2019) 30:1121–6. doi: 10.1093/annonc/mdz131 PMC6637370 30980071

[4] Planchard D Besse B Groen HJM Hashemi SMS Mazieres J Kim TM . Phase 2 study of dabrafenib plus trametinib in patients with BRAF V600E-mutant metastatic NSCLC: updated 5-year survival rates and genomic analysis. J Thorac Oncol Off Publ Int Assoc Study Lung Cancer. (2022) 17:103–15. doi: 10.1016/j.jtho.2021.08.011 34455067

[5] Weinstein IB Joe A . Oncogene addiction. Cancer Res. (2008) 68:3077–80. doi: 10.1158/0008-5472.CAN-07-3293 18451130

[6] Zhang YL Yuan JQ Wang KF Fu XH Han XR Threapleton D . The prevalence of EGFR mutation in patients with non-small cell lung cancer: a systematic review and meta-analysis. Oncotarget. (2016) 7:78985–93. doi: 10.18632/oncotarget.12587 PMC5346692 27738317

[7] Marchetti A Felicioni L Malatesta S Grazia Sciarrotta M Guetti L Chella A . Clinical features and outcome of patients with non-small-cell lung cancer harboring BRAF mutations. J Clin Oncol Off J Am Soc Clin Oncol. (2011) 29:3574–9. doi: 10.1200/JCO.2011.35.9638 21825258

[8] Cho BC Felip E Hayashi H Thomas M Lu S Besse B . MARIPOSA: phase 3 study of first-line amivantamab + lazertinib versus osimertinib in EGFR-mutant non-small-cell lung cancer. Future Oncol Lond Engl. (2022) 18:639–47. doi: 10.2217/fon-2021-0923 34911336

[9] Planchard D Jänne PA Cheng Y Yang JCH Yanagitani N Kim SW . Osimertinib with or without chemotherapy in EGFR-mutated advanced NSCLC. N Engl J Med. (2023) 389:1935–48. doi: 10.1056/NEJMoa2306434 37937763

[10] Hellyer JA White MN Gardner RM Cunanan K Padda SK Das M . Impact of tumor suppressor gene co-mutations on differential response to EGFR TKI therapy in EGFR L858R and exon 19 deletion lung cancer. Clin Lung Cancer. (2022) 23:264–72. doi: 10.1016/j.cllc.2021.09.004 34838441

[11] Rosell R González-Cao M Codony-Servat J Molina-Vila MA de Las Casas CM Ito M . Acquired BRAF gene fusions in Osimertinib resistant EGFR-mutant non-small cell lung cancer. Transl Cancer Res. (2023) 12:456–60. doi: 10.21037/tcr-22-2888 PMC10080327 37033354

[12] Dearden S Stevens J Wu YL Blowers D . Mutation incidence and coincidence in non small-cell lung cancer: meta-analyses by ethnicity and histology (mutMap). Ann Oncol. (2013) 24:2371–6. doi: 10.1093/annonc/mdt205 PMC3755331 23723294

[13] Pavan A Bragadin AB Calvetti L Ferro A Zulato E Attili I . Role of next generation sequencing-based liquid biopsy in advanced non-small cell lung cancer patients treated with immune checkpoint inhibitors: impact of STK11, KRAS and TP53 mutations and co-mutations on outcome. Transl Lung Cancer Res. (2021) 10:202–20. doi: 10.21037/tlcr-20-674 PMC7867770 33569305

[14] Nardo G Carlet J Marra L Bonanno L Boscolo A Dal Maso A . Detection of low-frequency KRAS mutations in cfDNA from EGFR-mutated NSCLC patients after first-line EGFR tyrosine kinase inhibitors. Front Oncol. (2020) 10:607840. doi: 10.3389/fonc.2020.607840 33520716 PMC7844327

[15] Owsley J Stein MK Porter J In GK Salem M O’Day S . Prevalence of class I-III BRAF mutations among 114,662 cancer patients in a large genomic database. Exp Biol Med Maywood NJ. (2021) 246:31–9. doi: 10.1177/1535370220959657 PMC7797994 33019809

[16] Razzaque MA Nishizawa T Komoike Y Yagi H Furutani M Amo R . Germline gain-of-function mutations in RAF1 cause Noonan syndrome. Nat Genet. (2007) 39:1013–7. doi: 10.1038/ng2078 17603482

[17] Niihori T Aoki Y Narumi Y Neri G Cavé H Verloes A . Germline KRAS and BRAF mutations in cardio-facio-cutaneous syndrome. Nat Genet. (2006) 38:294–6. doi: 10.1038/ng1749 16474404

[18] Sun M Wang X Xu Y Sun C Guo Y Qiu S . Combined targeting of EGFR and BRAF triggers regression of osimertinib resistance by using osimertinib and vemurafenib concurrently in a patient with heterogeneity between different lesions. Thorac Cancer. (2022) 13:514–6. doi: 10.1111/1759-7714.14295 PMC8807254 34962076

[19] Ribeiro MFSA Knebel FH Bettoni F Saddi R Sacardo KP Canedo FSNA . Impressive response to dabrafenib, trametinib, and osimertinib in a metastatic EGFR-mutant/BRAF V600E lung adenocarcinoma patient. NPJ Precis Oncol. (2021) 5:5. doi: 10.1038/s41698-021-00149-4 33580193 PMC7880994

[20] Meng P Koopman B Kok K Ter Elst A Schuuring E van Kempen LC . Combined osimertinib, dabrafenib and trametinib treatment for advanced non-small-cell lung cancer patients with an osimertinib-induced BRAF V600E mutation. Lung Cancer Amst Neth. (2020) 146:358–61. doi: 10.1016/j.lungcan.2020.05.036 32534795

